# Interaction among *COX*-2, *P2Y1* and *GPIIIa* gene variants is associated with aspirin resistance and early neurological deterioration in Chinese stroke patients

**DOI:** 10.1186/s12883-016-0788-8

**Published:** 2017-01-09

**Authors:** Xingyang Yi, Chun Wang, Qiang Zhou, Jing Lin

**Affiliations:** 1 Department of Neurology, People’s Hospital of Deyang City, No 173, North Taishan Road, Deyang, 618000 Sichuan China; 2 Department of Neurology, The Third Affiliated Hospital of Wenzhou Medical University, No 108 Wanson road, Ruan City, Wenzhou, 325200 Zhejiang China

**Keywords:** Ischemic stroke, Aspirin resistance, Genetic variants, Polymorphism, Early neurological deterioration

## Abstract

**Background:**

The effect of genetic variants on aspirin resistance (AR) remains controversial. We sought to assess the association of genetic variants with AR and early clinical outcomes in patients with acute ischemic stroke (IS).

**Methods:**

A total of 850 acute IS patients were consecutively enrolled. Platelet aggregation was measured before and after a 7–10 day aspirin treatment. The sequences of 14 variants of *COX-1, COX-2*, *GPIb, GPIIIa, P2Y1* and *P2Y12* were determined using matrix-assisted laser desorption/ionization time of flight mass spectrometry. Gene-gene interactions were analyzed using generalized multifactor dimensionality reduction (GMDR). The primary outcome was early neurological deterioration (END) within 10 days of admission. The secondary outcome was a composite of early recurrent ischemic stroke (ERIS), myocardial infarction (MI) and death within 10 days of admission.

**Results:**

175 (20.6%) patients were AR, 45 (5.3%) were aspirin semi-resistant, 121 (14.2%) developed END, 17 (0.2%) had ERIS, 2 (0.2%) died, and 6 (0.7%) had MI. Single locus analysis indicated that only rs1371097 was associated with AR. However, GMDR analysis indicated that the following three sets of gene-gene interactions were significantly associated with AR: rs20417CC/rs1371097TT/rs2317676GG; rs20417CC/rs1371097TT/rs2317676GG; rs20417CC/rs1371097CT/rs2317676AG. END occurred significantly more frequently in patients with AR or high-risk interactive genotypes. Moreover, AR and high-risk interactive genotypes were independently associated with END.

**Conclusion:**

Sensitivity of IS patients to aspirin and END may be multifactorial and is not significantly associated with a single gene polymorphism. Combinational analysis may useful for further insight into the genetic risks for AR.

## Background

Stroke is one of the leading causes of human mortality and disability [1]. Early neurological deterioration (END) and recurrent ischemic stroke (ERIS) are common and are associated with poor prognosis in acute ischemic stroke (IS) patients [2]. Aspirin is routinely used for the treatment of IS [3], and its use is associated with improved outcomes [2, 4]. However, recent studies have shown that aspirin resistance (AR) can lead to the failure of antiplatelet therapy [5], and is associated with frequent END and ERIS in patients with acute IS [6, 7]. Thus, further identification of risk factors for AR could improve the treatment of patients at risk of IS, enabling clinicians to choose more effective treatments.

Aspirin acts by inhibiting platelet cyclooxygenase enzymes (COX), preventing generation of thromboxane A2 (TXA2) from arachidonic acid (AA). TXA2 binds to glycoprotein coupled receptor (GPIIb/IIIa) leading to phospholipase C activation and platelet aggregation [8]. The platelet membrane receptors P2Y12 and P2Y1 play a key role in platelet aggregation, thrombosis and pharmacological targeting of these receptors [9]. Adenosine diphosphate (ADP) amplifies multiple signal pathways to activate platelets through autocrine and paracrine mechanisms, whereas inhibition of P2Y12 receptors prevents ADP-induced platelet activation [10]. P2Y12 antagonists have been shown to potently inhibit platelet activation [11]. Therefore, genetic variants of these signal molecules may contribute to AR.

A number of studies have assessed the association of AR with single nucleotide polymorphisms (SNPs) in *COX* and the above mentioned receptors genes. For example, polymorphisms in *COX-1, COX-2, GPIIIa, P2Y1*, *P2Y12* were reported to contribute to AR [12–17]. However, other studies, including a previous study published by this group, did not find aspirin responsiveness to be associated with these variants in patients with symptomatic vascular disease [18–20]. Thus, the role of SNPs in *COX-1, COX-2*, *GPIb, GPIIIa, P2Y1, P2Y12* in AR remains controversial [21]. These conflicting findings indicate that the contribution of genetic factors to AR may involve a complex network of mutations. It is possible that the effects of each locus alone may be too small to be detected in relatively small patient groups, and only specific combinations of multiple variants were found to significantly contribute to AR. Thus, investigating multiple gene-gene interactions using the generalized multifactor dimensionality reduction (GMDR) approach may be required to accurately characterize the genetic etiology of AR [22, 23]. However, no such studies investigating the effect of gene-gene interactions on AR have been reported.

We hypothesize that the interaction of particular genetic variants may contribute to AR and END in IS patients. In this study, we assessed the potential contribution of fourteen variants in six genes to AR and END in acute IS patients using GMDR analysis.

## Methods

### Ethics statement

The study protocol was approved by the Ethics Committee of the People’s Hospital of Deyang City and the Third Affiliated Hospital of Wenzhou Medical University. Written informed consent was obtained from each patient prior to study enrollment.

### Study population

We consecutively enrolled 850 acute IS patients between August 2010 and August 2014. These patients had their first IS and were admitted to the participating hospitals within 72 h of stroke onset. The inclusion criteria were: 1) age ≥ 40 years old; 2) IS was confirmed using brain magnetic resonance imaging; 3) stroke etiology was atherothrombotic or small artery disease subtype according to a previously described Acute Stroke Treatment classification system [24]; 4) National Institutes of Health Stroke Scale (NIHSS) score <15. Exclusion criteria were: 1) hemorrhagic stroke, or recurrent stroke; 2) aspirin allergy; 3) treatment combined aspirin and clopidogrel; 4) other etiologies of IS; 5) fever, infection, un consciousness at admission; 6) administration of other nonsteroidal anti-inflammatory drugs within 2 week prior to enrollment; 7) using low molecular weight heparin or heparin within 24 h of enrollment, or thrombolytic treatment; 8) carotid endoartectomy or carotid stent therapy before or during the follow-up period; 9) platelet count <100 × 10^9^/L or >450 × 10^9^/L; 10) asthma or severe cardiovascular, liver, or renal disease.

All patients received standard therapies [3, 25], including 200 mg/day aspirin (Bayer Healthcare Company Ltd., Beijing, China) for 14 days, and 100 mg/day thereafter. Data on various risk factors including age, gender, current smoking, history of diabetes mellitus and hypertension, were recorded. Fasting blood samples were tested for triglycerides (TG), total plasma cholesterol (TC), low-density lipoprotein cholesterol (LDL-C) and high-density lipoprotein cholesterol (HDL-C).

### Platelet aggregation test

Venous blood (3 mL) was drawn from each patient’s antecubital vein before and after 7–10 days of aspirin treatment. Platelet aggregation was measured by light transmittance aggregometry (LTA), as described previously [2, 5, 18]. A mean aggregation of ≥70% with 10 μM ADP and a mean aggregation of ≥20% with 0.5 mM AA after aspirin intake for 7 to 10 days were defined as AR. A mean aggregation of ≥70% with 10 μM ADP or a mean aggregation of ≥20% with 0.5 mM AA was defined as Aspirin semi-resistance (ASR). Patients with AR and those with ASR were pooled into an AR + ASR group [5, 18]. Otherwise, patients were considered aspirin sensitive (AS).

### Genotyping

A total of 14 variants in six genes, including *COX-1* (rs1236913, rs3842787), *COX-2* (rs689466, rs20417), *TXAS1* (rs194149, rs2267679, rs41708), *P2Y1* (rs701265, rs1439010, and rs1371097), *P2Y12* (rs16863323, rs9859538), and *GPIIIa* (rs2317676, rs11871251) were selected from the NCBI database (http://www.ncbi.nlm.nih.gov/SNP), based on the following criteria: (i) SNPs that have been assessed in previous studies [12–21]; (ii) SNPs with minor allele frequency >0.05; (iii) SNPs leading to amino acid changes.

Whole blood (3 mL) was drawn from the antecubital vein into a sterile tube containing ethylenediaminetetraacetic acid and stored at -80 °C. Genotypes for the 14 variants were assessed using a matrix-assisted laser desorption/ionization time of flight mass spectrometry as previously described [23]. Each allele was classified according to its effect on enzymatic function. For each gene, subjects were dichotomized *a priori* into two groups based on whether they possessed at least one mutant allele.

### Assessment of clinical outcomes

The clinical outcomes of IS patients were assessed within the first 10 days of admission. The primary outcome was END, defined as an increase in NIHSS score of ≥ 4 points within 10 days of admission, after exclusion of hemorrhagic transformation of infarct or a new infarct in another vascular territory as previously described [26]. The secondary outcome was a composite of ERIS, myocardial infarction (MI), and death within 10 days of admission. ERIS was defined as a sudden and new focal neurologic deficit of vascular origin lasting at least 24 h, indicated in a diffusion-weighted image [26]. Death was defined as all-cause mortality.

### Statistical analysis

All statistical analyses were performed using SPSS 16.0 (SPSS Inc., Chicago, IL, USA). Deviation of Hardy-Weinberg equilibrium for genotype frequencies was analyzed by *χ*2-test. Difference in genotype frequencies between the AR + ASR group and AS group were also compared using the *χ*2-test. Baseline and clinical characteristics were compared using the *χ* ^2^ test or Fisher exact test (categorical variables) and the Student’s *t* test (continuous variables). Gene–gene interactions were assessed using the GMDR program (β version 0.7, www.healthsystem.virginia.edu/internet/addiction-genomics/Software) as previously reported [22, 23].

Significant independent predictors of AR were analyzed by logistic regression analysis. The relative risk of a genotype with AR was expressed as odds ratio (OR) with 95% confidence interval (CI). The Cox proportional-hazards model was used to describe the risk for primary outcome and calculate the hazard ratio (HR) with 95% CI. The variables entered into the model were the variables that differed significantly between the AR + ASR group and AS group. All tests were two-sided, and the threshold level of *P* < 0.05 indicated statistical significance.

## Results

### Occurrence of AR in acute IS patients

Of the 850 patients taking aspirin, AR was detected in 175 (20.6%), ASR was detected in 45 (5.3%), and AS was detected in 630 (74.1%). Diabetes mellitus, LDL-C and fasting glucose levels and female gender were significantly associated with ASR + AR in univariate analysis (*P* = 0.02, Table 1).

**Table 1:** Comparison of clinic characteristics between AS and ASR or AR patients

Parameter	AR + ASR	AS	*P*-value
	*n* = 220	*n* = 630	
Age (years)	70.8 ± 12.76	70.01 ± 11.35	0.94
Gender (female, %)	122 (55.45)	285 (45.24)	0.02
Body mass index (kg/m^2^)	23.85 ± 3.34	23.92 ± 3.32	0.99
Current smoking (n, %)	66 (30.00)	182 (28.89)	0.62
Hypertension (n, %)	157 (71.36)	439 (69.68)	0.89
Diabetes (n, %)	79 (35.91)	112 (17.78)	<0.001
Previous MI (n, %)	7 (3.2)	14 (2.2)	0.72
NIHSS score at enrollment	5.91 ± 1.81	5.87 ± 1.86	0.89
TC (mmol/L)	5.32 ± 1.18	5.29 ± 1.76	0.26
TG (mmol/L)	1.80 ± 0.83	1.79 ± 0.90	0.96
HDL-C (mmol/L)	1.32 ± 0.32	1.31 ± 0.33	0.85
LDL-C (mmol/L)	3.13 ± 0.92	2.93 ± 0.86	<0.001
Fasting glucose (mmol/L)	7.21 ± 2.12	6.43 ± 1.86	<0.001
Platelet count (×10^9^/L)	193.01 ± 28.62	196.42 ± 30.46	0.79
Stroke subtype			
Atherothrombotic (n, %)	135 (61.36)	384 (60.95)	0.86
Small artery disease (n, %)	85 (38.64)	246 (39.05)	0.86

*AR* aspirin resistance, *ASR* aspirin semi-resistance, *AS* aspirin sensitivity, *MI* myocardial infarction, *TC* total cholesterol, *LDL-C* low-density lipoprotein cholesterol, *HDL-C* high-density lipoprotein cholesterol, *TG* triglycerides, *NIHSS* National Institutes of Health Stroke Scale

### Association of SNPs with AR

Genotype distributions for the 14 variants were in accordance with Hardy–Weinberg equilibrium (*P* > 0.05). The frequency of TT + CT genotypes for rs1371097 was significantly higher in the AR + ASR group than in AS group (*P* = 0.01, Table 2). However, the single-locus analytical approach did not identify any differences in the frequencies of other variant genotypes between the two groups (all *P* > 0.05, Table 2).

**Table 2:** Association of SNPs with responses of aspirin in IS patients (%)

	AR + ASR	AS	*P*-value
	*n* = 220	*n* = 630	
*COX-1* (rs1236913)			
CC	198 (90.0)	567 (90.0)	0.99
CT + TT	22 (10)	63 (10.0)	
*COX-1* (rs3842787)			
CC	171 (77.7)	504 (80.0)	0.76
CT + TT	49 (22.3)	126 (20.0)	
*COX-2* (rs689466)			
AA	63 (28.6)	180 (29.6)	0.89
AG + GG	157 (71.4)	450 (71.4)	
*COX-2* (rs20417)			
GG	142 (64.5)	441 (70.0)	0.26
GC + CC	78 (35.5)	189 (30.0)	
*TXAS1* (rs194149)			
GG	75 (34.1)	190 (30.2)	0.42
AG + AA	145 (65.1)	440 (69.8)	
*TXAS1* (rs2267679)			
TT	178 (80.9)	522 (82.9)	0.53
CC + CT	42 (19.1)	108 (17.1)	
*TXAS1* (rs41708)			
GG	136 (61.8)	373 (59.2)	0.72
TT + GT	84 (38.2)	257 (40.8)	
*P2Y1* (rs701265)			
AA	116 (52.7)	341 (54.1)	0.48
AG + GG	104 (47.3)	289 (45.9)	
*P2Y1* (rs1439010)			
AA	114 (51.8)	346 (54.9)	0.32
AG + GG	106 (48.2)	284 (45.1)	
*P2Y1* (rs1371097)			
CC	106 (48.2)	373 (59.2)	0.01
TT + CT	114 (51.8)	257 (40.8)	
*P2Y12* (rs16863323)			
CC	48 (21.8)	158 (25.1)	0.21
TT + CT	172 (78.2)	472 (74.9)	
*P2Y12* (rs9859538)			
GG	154 (70.0)	472 (74.9)	0.16
AG + AA	66 (30.0)	158 (25.1)	
*GPIIIa* (rs2317676)			
AA	132 (60.0)	404 (64.1)	0.24
AG + GG	88 (40.0)	64 (35.9)	
*GPIIIa* (rs11871251)			
AA	77 (35.0)	190 (30.2)	0.51
AG + GG	143 (65.0)	440 (69.8)	

### Gene-gene interaction and its association with AR

We next used the GMDR method to investigate the association of the 14 variants high-order interactions with AR + ASR. With covariate adjustments, the best model for AR + ASR was rs20417, rs1371097 and rs2317676, which scored 10/10 for cross-validation consistency and 9/10 for the sign test (*P* = 0.016). The one-locus model was computed for each variant and the prediction accuracy of these one-locus models was 0.5634, 0.5395 and 0.5237 (for rs20417, rs1371097, and rs2317676, respectively), and a minimum *P* value was 0.9428. The significance of this interaction was then confirmed using a permutation test (*P* = 0.019), indicating that together these three genetic variants significantly contribute to ASR + AR. Further analysis demonstrated that, in comparison to patients harboring wild-type genotype rs20417GG, rs1371097CC, and rs2317676AA, the gene interactions contributing most significantly to this model were rs20417CC, rs1371097TT, and rs2317676GG; rs20417CC, rs1371097TT, and rs2317676GG/AG; rs20417CC, rs1371097CT, and rs2317676AG (Table 3). These data demonstrated that interaction of the three genetic variants contributed to ASR + AR in IS patients.

**Table 3:** Association between cerebral infarction and genotype combinations

rs20417	GG	CC	CC	CC	GC	CC, GC	CC	CC, GC
rs1371097	CC	TT	TT	CT	CT	TT	TT, CT	TT, CT
rs2317676	AA	GG	AG, GG	AG	AG	GG	GG	GG, AG
OR	1 ^a^	2.72	1.91	2.28	1.31	1.08	1.11	1.05
95% CI	-	1.18-6.86	1.07–3.84	1.13–5.33	0.98–3.26	0.72–1.85	0.62–2.21	0.64–1.75
*P* value	-	0.004	0.034	0.025	0.087	0.257	0.452	0.678

*OR* odds ratio

^a^ The low-risk genotype for each genetic factor was used as the reference OR

### Risk factors for ASR + AR

The relative risk these three variants conferred was considered an interactive variable, with high-risk assigned as one and low-risk assigned as zero. Logistic regression indicated that high-risk interactive variables were significant independent predictors of ASR + AR (OR = 2.35, 95% CI: 1.87–6.86, *P* = 0.002) after adjusting for other covariates (Table 4).

**Table 4:** Logistic regression analysis of the significant independent predictors of ASR + AR

Risk factor	*OR*	95% CI	*P* value
Female	0.86	0.69–1.34	0.232
Diabetes mellitus	2.02	1.14–4.23	0.023
High LDL-C	0.97	0.92–2.87	0.102
High blood glucose	1.02	0.93–3.42	0.086
Rs1371097TT/CT	0.92	0.84–2.34	0.124
High-risk interactive variable	2.35	1.87–6.86	0.002

*AR* aspirin resistance, *ASR* aspirin semi-resistance, *OR* odds ratio, *CI* confidence intervals, *LDL-C* low-density lipoprotein cholesterol

### Clinical outcome

Of the 850 patients enrolled, 121 (14.2%) developed END, 17 (0.2%) had ERIS, 2 (0.2%) died, and 6 (0.7%) had MI within 10 days of admission. The frequency of END was higher in the AR + ASR group than that in AS group (Table 5). However, the frequencies of ERIS, MI and death did not differ significantly between the two groups. Clinically, patients with END were older (70.8 ± 10.9 vs. 66.9 ± 11.7, *P* < 0.001), had higher glucose levels at baseline (7.6 ± 2.2 vs. 6.3 ± 1.9, *P* < 0.001), and had a higher prevalence of diabetes mellitus (54.5% [66/121] vs. 17.1% [125/729], *P* < 0.001) compared with patients without END. Furthermore, END was more common in patients carrying high-risk interactive genotypes than those with no such interactive genotypes (41.2% [61/148] vs. 8.5% [60/702], *P* < 0.001). Cox regression analysis revealed that high-risk interactive genotypes (HR: 2.47, 95% CI, 1.42–7.8, *P* < 0.01), AR + ASR (HR: 2.04, 95% CI, 1.36–6.25, *P* = 0.01), and high glucose level (HR: 1.56, 95% CI, 1.06–4.78, *P* = 0.03) were independent risk factors for END (Table 6).

**Table 5:** Association of aspirin resistance with clinical outcomes

	AR + ASR	AS	*P* value
	*n* = 220	*n* = 630	
END (n, %)	69 (31.4)	52 (8.3)	<0.001
ERIS (n, %)	5 (2.3)	12 (1.9)	0.74
MI (n, %)	2 (0.9)	4 (0.6)	0.83
Death (n, %)	0 (0.0)	2 (0.3)	0.82

*AR* aspirin resistance, *ASR* aspirin semi-resistance, *AS* aspirin sensitivity, *END* early neurological deterioration, *ERIS* early recurrent ischemic stroke, *MI* myocardial infarction

**Table 6:** Cox regression analysis of risk factors for END

Factor	Hazard ratio	95% CI	*P* value
Age	0.86	0.69–1.42	0.82
Diabetes mellitus	1.18	0.97–2.85	0.08
High fasting glucose	1.56	1.06–4.78	0.03
AR + ASR	2.04	1.36–6.25	0.01
High-risk interactive genotypes	2.47	1.42–7.84	<0.01

*END* early neurological deterioration, *AR* aspirin resistance, *ASR* aspirin semi-resistance, *CI* confidence intervals

### Effect of genotypes on platelet aggregation in both pre- and post-aspirin treatment

Platelet aggregation did not differ significantly among the 14 variants, or between the patients with and without high-risk interactive genotypes at admission. Platelet aggregation also did not differ among the 14 variants post-aspirin treatment. However, after aspirin treatment platelet aggregation was significantly higher in patients carrying high-risk interactive genotypes than those without high-risk interactive genotypes (*P* < 0.001, Table 7).

**Table 7:** Effect of genotypes on platelet aggregation of both pre- and post-aspirin treatment (%)

	Pre-aspirin aggregation	Platelet	Post-aspirin aggregation	Platelet
	AA- induced	ADP-induced	AA- induced	ADP-induced
*COX-1* (rs1236913)				
CC (*n* = 765)	88.4 ± 15.7	89.2 ± 14.7	18.9 ± 9.2	66.8 ± 12.8
CT + TT (*n* = 85)	89.2 ± 14.6	89.7 ± 13.2	19.4 ± 8.8	67.5 ± 10.8
*P*-value	0.65	0.73	0.63	0.57
*COX-1* (rs3842787)				
CC (*n* = 675)	87.9 ± 16.3	88.6 ± 14.7	19.2 ± 8.4	67.7 ± 11.5
CT + TT (*n* = 175)	89.7 ± 13.6	90.1 ± 15.8	19.0 ± 7.8	66.6 ± 12.3
*P*-value	0.15	0.21	0.86	0.28
*COX-2* (rs689466)				
AA (*n* = 243)	88.9 ± 16.3	89.2 ± 12.8	20.1 ± 10.2	66.8 ± 13.2
AG + GG (*n* = 607)	89.7 ± 14.1	90.3 ± 16.9	18.9 ± 9.8	67.2 ± 11.5
*P*-value	0.51	0.36	0.12	0.67
*COX-2* (rs20417)				
GG (*n* = 583)	87.9 ± 15.4	88.3 ± 15.2	18.2 ± 8.6	65.9 ± 13.4
GC + CC (*n* = 267)	89.7 ± 15.7	89.6 ± 14.6	19.5 ± 9.6	67.1 ± 14.9
*P*-value	0.13	0.26	0.34	0.07
*TXAS1* (rs194149)				
GG (*n* = 265)	90.2 ± 16.7	89.4 ± 12.9	19.1 ± 7.6	67.9 ± 12.2
AG + AA (*n* = 585)	88.6 ± 17.5	88.9 ± 15.6	18.7 ± 6.7	68.4 ± 13.8
*P*-value	0.22	0.64	0.47	0.64
*TXAS1* (rs2267679)				
TT (*n* = 700)	88.9 ± 17.7	89.1 ± 14.9	19.3 ± 8.9	66.5 ± 14.2
CC + CT (*n* = 150)	90.3 ± 15.6	90.2 ± 16.4	18.4 ± 8.2	67.9 ± 15.5
*P*-value	0.33	0.44	0.19	0.31
*TXAS1* (rs41708)				
GG (*n* = 509)	88.8 ± 13.6	88.9 ± 15.2	18.8 ± 7.2	68.1 ± 12.5
TT + GT (*n* = 341)	90.1 ± 17.8	89.4 ± 13.8	19.3 ± 8.4	67.6 ± 11.1
*P*-value	0.18	0.63	0.41	0.54
*P2Y1* (rs701265)				
AA (*n* = 457)	88.9 ± 15.3	88.7 ± 16.1	19.2 ± 7.7	67.8 ± 13.2
AG + GG (*n* = 393)	89.6 ± 17.7	89.5 ± 14.2	18.4 ± 8.1	68.5 ± 14.4
*P*-value	0.54	0.42	0.15	0.44
*P2Y1* (rs1439010)				
AA (*n* = 460)	90.3 ± 16.8	90.1 ± 16.6	19.1 ± 6.8	66.9 ± 13.6
AG + GG (*n* = 390)	88.5 ± 15.2	89.0 ± 15.3	18.7 ± 7.4	67.7 ± 12.2
*P*-value	0.11	0.31	0.42	0.34
*P2Y1* (rs1371097)				
CC (*n* = 479)	89.2 ± 15.6	87.9 ± 16.2	18.8 ± 7.2	68.1 ± 12.5
TT + CT (*n* = 371)	88.6 ± 14.1	89.2 ± 15.6	19.3 ± 8.4	67.6 ± 11.1
*P*-value	0.57	0.21	0.31	0.62
*P2Y12* (rs16863323)				
CC (*n* = 206)	87.9 ± 16.4	88.8 ± 16.4	18.9 ± 7.6	67.2 ± 13.2
TT + CT (*n* = 644)	89.9 ± 18.7	89.2 ± 14.5	18.7 ± 8.2	67.8 ± 12.3
*P*-value	0.14	0.76	0.73	0.51
*P2Y12* (rs9859538)				
GG (*n* = 626)	88.5 ± 16.5	88.9 ± 14.3	18.4 ± 8.7	66.7 ± 11.
AG + AA (*n* = 224)	89.9 ± 16.8	90.1 ± 16.7	19.1 ± 7.9	67.5 ± 13.6
*P*-value	0.28	0.31	0.33	0.41
*GP*III*a* (rs2317676)				
AA (*n* = 536)	89.5 ± 15.2	87.9 ± 13.7	19.2 ± 8.2	68.3 ± 14.2
AG + GG (*n* = 314)	88.2 ± 15.7	89.4 ± 17.8	18.5 ± 7.1	67.4 ± 13.3
*P*-value	0.32	0.23	0.26	0.36
GPIIIa (rs11871251)				
AA (*n* = 267)	89.2 ± 12.6	89.8 ± 16.6	19.4 ± 8.7	66.9 ± 15.7
AG + GG (*n* = 583)	88.6 ± 14.9	89.2 ± 14.3	18.6 ± 7.5	67.5 ± 14.4
*P*-value	0.53	0.56	0.19	0.49
High-risk interactive genotypes				
Yes (*n* = 147)	90.6 ± 18.4	90.3 ± 17.5	30.3 ± 9.2	79.9 ± 12.2
No (*n* = 703)	88.8 ± 16.7	88.7 ± 13.8	15.6 ± 6.8	62.3 ± 11.1
*P*-value	0.28	0.27	< 0.001	< 0.001

## Discussion

In the present study, we used the GMDR approach to investigate the potential contribution of fourteen variants in six genes to AR and END in 850 patients with acute IS. We found that the prevalence of AR and ASR was 20.6 and 5.3%, respectively, and that diabetes mellitus was associated with AR. These findings were in accordance with previous studies [5, 18, 27]. Further, aside from variant *P2Y1* (rs1371097), we found no significant differences in the distribution of the other variants between the AR + ASR group and AS group. However, a high-risk interactive genotype, involving rs20417, rs1371097, rs2317676, was independently associated with AR and END.

The mechanisms underlying AR have not yet been fully elucidated. Previous studies have suggested that AR is correlated with gender, age, absorption or metabolic dysfunction, drug compliance, drug interactions, diabetes mellitus, aspirin dose, cholesterol levels, and inflammatory markers [28, 29]. Furthermore, the biosynthesis of TXA2 and alternative pathways involved in platelet activation, including ADP, collagen, epinephrine and thrombin, have been implicated in AR [27, 30], suggesting that multiple factors contribute to AR. Our results indicate that female gender, LDL-C, diabetes mellitus and fasting glucose are significantly associated with AR. Chronic hyperlipidemia plays a key role in platelet activation both in vivo [31] and in vitro [32]. Davi et al. observed increased in vivo formation of 8-epi-prostaglandin F2 alpha in chronic hyperlipidemia that correlated with enhanced urine 11-dehydro-thromboxane B2 production [33]. This may indicate an aspirin-insensitive mechanism that links lipid peroxidation to amplification of platelet activation. Diabetes mellitus or high fasting glucose is associated with hyperlipidemia, platelet dysfunction, chronic inflammation and endothelial dysfunction, which impair aspirin responsiveness [34]. In diabetic patients, other mechanisms that may be responsible for reduced aspirin responsiveness include increased calcium or esterase levels, circulating ADP, and platelet turnover, P2Y12 receptor expression, upregulation of other platelet activation pathways [35, 36]. Therefore, for IS patients with diabetes or hyperlipidemia intensive antiplatelet therapy may be important.

With the advances in next generation sequencing and genetic association analysis, accumulating evidence has suggested that genetic factors may be associated with AR. *COX* and its receptors are involved in platelet activation and aggregation, and are inhibited by aspirin. Polymorphisms in *COX*-2 rs20417 and *COX-1* C50T have been reported to be associated with impaired aspirin responsiveness [13, 14]. However, in both a previous study [18] and this study, these polymorphisms were not found to be associated with AR in acute IS patients. P2Y12 and P2Y1 are platelet membrane receptors and play a key role in platelet aggregation, thrombosis and the pharmacology of antiplatelet therapy [9]. Grinshtein et al.[17] reported that AR was associated with *P2Y12* and *P2Y1* polymorphisms; but Goodman et al.[15] did not find these polymorphisms to be associated with AR. The GPllb/llla receptor is a major regulator of platelet aggregation. GPllb/llla bind fibrinogen and cross-link adjacent platelets. Some studies have suggested that *GPllb/llla* variants are associated with AR [15, 37], whereas other studies have not found such association [38]. This lack of consistency in the literature makes it difficult to define the effects of these gene variants on aspirin responsiveness. The potential reasons for the conflicting results may include divergent ethnicity-specific genetic profiles, population stratification, sample sizes [39]. These controversial results may also reflect the complexity of mechanisms by which genetic factors contribute to AR. Therefore, it may be crucial to analyze the effects of gene-gene interactions on AR risk. In this study, GMDR analysis found that a high-risk interactive genotype, involving rs20417, rs1371097, rs2317676, was associated with AR and END. The mechanism by which the three variants interact is unclear. The three genes encode COX enzymes and platelet activation and aggregation receptors which modulate aspirin pharmacodynamics and pharmacokinetics, a principal component of the aspirin response. Our results demonstrated that AA or ADP induced significantly more platelet aggregation in patients carrying high-risk interactive genotypes than those without these genotypes. We speculate that the interaction of rs20417, rs1371097 and rs2317676 could potentially cause high platelet aggregation in these individuals, thus increasing risk of AR and END. However, further studies will be required to investigate the mechanisms by which these gene interactions impair aspirin responsiveness.

In this study, the frequency of END was 14.2%, and high-risk interactive genotypes and AR were independently associated with END. While the mechanisms underpinning END have not been fully understood. Thrombosis is one of the causes of ischemic stroke, and platelet activation plays a key role in thrombosis [40], IS and END pathogenesis [26, 41]. AR and high-risk interactive genotypes may reduce inhibition of platelet activation [7], leading to END. Thus, for IS patients with AR or high-risk interactive genotypes antiplatelet therapy may be important.

Recurrent ischemic stroke is common in IS patients. In this study, the frequencies of ERIS, MI and death did not significantly differ between the AR + ASR group and AS group. Furthermore, we found that high-risk interactive genotypes did not increase the risk of recurrent stroke.

Our conclusions are limited by the scope of this particular study. Firstly, we enrolled a relatively small sample size, including only two centers, and the follow up period was relatively short. Our findings will need be validated in larger, multi-center studies that follow patients for longer. Secondly, although we genotyped multiple functional variants in known aspirin-relevant genes, some rare functional variants may have been overlooked; thus, we are not able to exclude the role of other polymorphisms in the regulation of AR. Finally, although the main aim of this study was to assess the occurrence of AR, and the association of genetic variants with AR and early clinical outcomes in acute ischemic stroke, we did not include the relevant negative control to allow the association of these gene variants with ischemic stroke to be analyzes. We therefore could not eliminate the possibility that these gene variants interact to contribute to acute stroke themselves. Thus, it will be essential to include a non-stroke group taking aspirin in future studies.

## Conclusion

We assessed the contribution of fourteen variants from six genes with AR and END in acute IS patients using the GMDR approach. We found that combinations of rs20417CC, rs1371097TT, and rs2317676GG; rs20417CC, rs1371097TT, and rs2317676GG/AG; rs20417CC, rs1371097CT, and rs2317676AG were associated with a higher risk of AR in IS patients. Moreover, AR and high-risk interactive genotypes were independently associated with END. Our findings suggest that the genetic basis for AR is complex, and that genotyping of multiple relevant genes may be necessary to predict AR risk and manage IS patients.

## Abbreviations

AA: Arachidonic acid
ADP: Adenosine diphosphate
AR: Aspirin resistance
AS: Aspirin sensitive
ASR: Aspirin semi-resistance
CI: Confidence interval
COX: Cyclooxygenase
END: Early neurological deterioration
ERIS: Early recurrent ischemic stroke
GMDR: Generalized multifactor dimensionality reduction
HR: Hazard ratio
HT: Hemorrhagic transformation
IS: Ischemic stroke
LDL-C: High-density lipoprotein cholesterol
LDL-C: Low-density lipoprotein cholesterol
LTA: Light transmittance aggregometry
MI: Myocardial infarction
NIHSS: National Institutes of Health Stroke Scale
OR: Odds ratio
SNPs: Single nucleotide polymorphisms
TC: Total cholesterol
TG: Triglycerides
TOAST: Trial of ORG 10172 in the Acute Stroke Treatment
TXA2: Thromboxane A2
